# Evaluation of the performance of Abbott Panbio™ COVID-19 antigen rapid diagnostic test for the detection of severe acute respiratory syndrome coronavirus 2 at Harar, Eastern Ethiopia

**DOI:** 10.3389/fmed.2023.1135027

**Published:** 2023-05-31

**Authors:** Zelalem Teklemariam, Dereje Feleke, Abdusemed Abdurahman, Astawus Alemayehu, Abebaw Demissie, Asefa Tufa, Nebiyu Sherefa, Abdusemed Mohammed, Mussie Brhane, Kasahun Bogale

**Affiliations:** ^1^School of Medical Laboratory Sciences, College Health and Medical Sciences, Haramaya University, Harar, Ethiopia; ^2^Department of Health Informatics, Harar Health Science College, Harar, Ethiopia; ^3^Harari Health Research and Regional Laboratory, Department of Microbiology, Harar, Ethiopia; ^4^Department of Public Health, Harar Health Science College, Harar, Ethiopia; ^5^Department of Anesthesia, Harar Health Science College, Harar, Ethiopia; ^6^Department of Field Epidemiology, Harari Regional Health Bureau, Harar, Ethiopia; ^7^Department of Health Economics, Harari Regional Health Bureau, Harar, Ethiopia; ^8^Department of Midwifery, Harar Health Science College, Harar, Ethiopia; ^9^Hararghe Health Research Laboratory, Haramaya University, Harar, Ethiopia; ^10^School of Medical Laboratory Sciences, College of Health and Medical Science, Haramaya University, Harar, Ethiopia

**Keywords:** RT-PCR, SARS-CoV-2, sensitivity, specificity, Harar, Eastern Ethiopia, Rapid diagnostic test, Panbio™

## Abstract

**Background:**

Rapid antigen tests can help in the effective isolation of symptomatic cases and the systematic tracing of close contacts. However, their reliability must be validated before implementing them widely.

**Methods:**

A cross-sectional study was conducted on 236 COVID-19-suspected patients visiting four different health institutions in Harari Regional State, Harar, Eastern Ethiopia, from June to July 2021. Two nasopharyngeal samples were collected and processed by the Panbio™ Ag-RDT kit and qRT-PCR. The collected data were analyzed using SPSS version 25.0.

**Results:**

The Panbio tests had a sensitivity of 77.5% (95% CI: 61.6–89.2%) and a specificity of 98.5% (95% CI: 95.6–99.7%). It also had a positive predictive value of 91.2% (95% CI: 76.9–96.9%), a negative predictive value of 95.5% (95% CI: 92.3–97.4%), and a kappa of 0.81 (95% CI: 0.7–0.9). The test had a sensitivity of 94.4%, 100%, 100%, and 90% in the samples collected from patients within the 1–5 days post-onset of COVID-19 signs and symptoms, of age group ≤18 years old, with cycle threshold values of <20, and with household contact, respectively.

**Conclusion:**

This test can be used as point-of-care testing for the diagnosis of symptomatic patients with short clinical courses and contact with patients in households.

## Background

Coronavirus disease 2019 or COVID-19 is caused by severe acute respiratory syndrome coronavirus 2 (SARS-CoV-2) (1). The virus has a range of manifestations, which can be asymptomatic, mild, and severe disease with fever to pneumonia. It also resulted in a huge number of deaths worldwide (2).

Rapid detection, effective isolation of symptomatic cases, and systematic tracing of close contacts are paramount to reducing the spread of SARS-CoV-2 in the community. The diagnosis of COVID-19 is confirmed by the detection of SARS-CoV-2 using real-time reverse transcription polymerase chain reaction (qRT-PCR) of nasopharyngeal or oropharyngeal samples from individuals suspected of the disease or having a history of contact with infected cases. The qRT-PCR assay is laborious and time-consuming, requiring specialized instruments, supplies, and expertise. Many countries have faced shortages in the supply of qRT-PCR reagents for the diagnosis of SARS-CoV-2 infection (3, 4).

Rapid diagnostic tests (RDT) of SARS-CoV-2 detect the antigens rapidly from nasal, nasopharyngeal and salivary, and other respiratory secretions. It is less expensive, simple to perform and to be interpreted at the point of care, and can be applied with minimal training. The test improves the turnaround time. It does not require specific equipment unlike nucleic acid amplification tests (NAATs). Moreover, the recently launched antigen assay appeared to be better correlated with patient infectiousness than qRT-PCR (4–6).

A number of SARS-CoV-2-RDT were developed worldwide with variable diagnostic performance in different settings (7, 8). However, there is no published report on the diagnostic performance of RDT in the eastern part of Ethiopia. Therefore, this study attempted to determine the diagnostic performance of the Panbio^™^ COVID-19 Antigen Rapid Test Device (Panbio Ag RDT) (Abbott) for detecting SARS-CoV-2 in nasopharyngeal swab specimens.

## Materials and methods

### Study area, design, and period

This cross-sectional study was conducted in Harari National Regional state, Harar town, which is one of the Ethiopian states located 515 km east of Addis Ababa. The region had four hospitals (three governmental and one private) and four health centers (8). The study was conducted from June to July 2021 on 236 patients attending outpatient departments of two Hospitals (Jugel and Harar General Hospitals) and two primary healthcare centers (Jinella and Arategna Health Centers) in the town, who had clinical symptoms related to COVID-19 or asymptomatic close contact with confirmed COVID-19 patients and had indications for qRT-PCR (MFG030011) testing, according to WHO criteria (1). The study areas were selected purposefully where the majority of patients suspected of SARS-CoV-2 infection seek SARS-CoV-2 testing. The prevalence of SARS-CoV-2 in the region was 17% during the data collection period (9).

### Data collection method

The following data collection approaches were used.

#### Face-to-face interview

Data were collected from each participant by a medical laboratory technologist using a structured questionnaire that was adapted from the standard laboratory request form prepared by Ethiopian Public Health Institutes for community and health facility surveillance of SARS-CoV-2 in Ethiopia. The questionnaire used to collect information on sociodemographic data, the presence or absence of COVID-19 symptoms, the number of days since the onset of symptoms, co-morbidities, qRT-PCR results, including cycle threshold (Ct) value, and the Panbio^™^ COVID-19 Ag-RDT.

#### Nasopharyngeal (NP) swab collection and the detection of SARS-CoV-2

Two NP samples were collected from each participant by a trained laboratory technologist using flocked swabs, following appropriate safety precautions. The first NP sample collected was tested using the Panbio^™^ Ag-RDT kit at a health facility without storage according to the manufacturer’s instructions. The Panbio^™^ COVID-19 Antigen is a membrane strip that is pre-coated with the immobilized anti-SARS-CoV-2 antibody on the test line and mouse monoclonal anti-chicken IgY on the control line. The tests were reported as positive when both control (C) and test (T) lines were visible, while negative when only the control (C) line is present, and invalid when no control (C) line is seen (10).

The second collected NP sample was placed in 3 mL of Universal Transport Medium (Becton Dickinson, Sparks, MD, United States) and transported at low-temperature conditions using an ice bag to Harari Health Research Laboratory (HHRL) for qRT-PCR testing. In HHRL, ribonucleic acid (RNA) was extracted using a QIAamp Viral kit (QIAGEN company). Amplification and detection of the Open Reading Frame (ORF) 1ab gene of SARS-CoV-2 were performed at the Beijing Genomics Institute (BGI Park, No. 21 Hongan 3rd Street, Yantian District, Shenzhen518083, China) using a QuantStudio 7 Flex qRT-PCR machine (Applied Biosystems, United States). Results were interpreted as positive SARS-CoV-2 if the standard curve at the fluorescein amidites (FAM) dye channel was S-shape (sigmoidal curve) with a cycle threshold (Ct) value of not higher than 38. The result was reported as negative SARS-CoV-2 if the standard curve at the FAM channel was not S-shape with a Ct value of zero or no data available while the Ct value at the VIC dye channel not higher than 32 (11).

### Quality control

All data collectors received quality control training about data and sample collection tools. Sample collection and processing were performed following standard operating procedures. In qRT-PCR, an extraction control (MOC) was used on each run to check for any contamination during each batch of extraction. Blank control and positive controls were tested during each experiment (11).

### Data analysis

Data were cleaned and analyzed using SPSS version 25.0 (SPSS, Chicago, IL, United States). Comparing the RT-PCR results to the gold standard, specificity, sensitivity, positive predictive value (PPV), and negative predictive value (NPV) with 95% confidence intervals (95% CI) were calculated. The performance evaluation of RDT against qRT-PCR was conducted by comparing the duration of signs and symptoms in days, age groups of study participants, history of contact with confirmed SARS-CoV-2 patients, the source of contact, and Ct value. Agreement between techniques was evaluated using Cohen’s kappa score (12). Statistical significance was defined as *p* < 0.05.

### Ethical consideration

This study protocol was reviewed and approved by Harari Health Bureau’s Ethical and Research Review Board. Each study participant obtained detailed information about the study. Voluntary written and signed consent was obtained from each study participant or their parent for children.

## Results

### Characteristics of the study participants

In this study, there were 236 participants, and the mean and standard deviation of their ages were 33.2 ± 15 years. The majority of them were older than 18 years (85.2%) and were male (52.4%). Most of the study participants (199, 84.3%) had at least one sign/symptom of COVID-19. Cough was the most common sign. On the other hand, approximately 25 (10.6%) of the study participants had co-morbidity. Hypertension was the most common co-morbidity. The mean and standard deviation of the duration of onset of COVID-19 signs and symptoms were 4.2 ± 2.8 days and ranges from 1 to 15 days. The major reason for current testing was suspected SARS-CoV-2 (72.9%) (Table 1).

**Table 1:** Characteristics of study participants providing nasopharyngeal and throat swabs for detection of SARS-CoV-2 qRT-PCR from a selected health facility in Harari Regional state, Harar, Eastern Ethiopia, from August to October 2021 (*n* = 236).

Variables	Category	Frequency
Age category	≤18	35 (14.8%)
	>18	201 (85.2%)
Sex	Male	126 (53.4)
	Female	110 (46.6)
Occupational status	Unemployed	17 (7.2%)
	Government employee	72 (30.5%)
	Private employee	73 (30.9%)
	Student	42 (17.8%)
	Others*	32 (13.6%)
Signs or symptoms of COVID-19	Cough	169 (71.8)
	Fever	113 (47.9)
	Shortness of breath	49 (20.8)
	Fatigue	34 (14.4)
	Sore throat	47 (19.9)
	Headache	52 (2202)
	Loss of smell	13 (5.5)
	Loss of taste	16 (6.8)
	Joint pain	7 (3.0)
Co-morbid condition	Diabetes mellitus	9 (3.8)
	Hypertension	13 (5.5)
	HIV	2 (0.8)
	Chronic respiratory diseases	4 (1.7)
	Chronic cardiac disease	3 (1.3)
	Pregnancy	7 (3.0)
Reason of testing	Suspected	172 (72.9)
	Contact with confirmed cases	64 (27.1)

*Other: house wife, daily labourer and no job; HIV: human Immuno deficiency virus.

A total of 64 patients had contact with confirmed cases of SARS-CoV-2 and 29 (45.3%) of them had at least one sign/symptom of COVID-19. A total of 47 (73.4%) and 17 (26.6%) of the study participants had contact with confirmed cases in their households and other places (workplace, social contact, and other), respectively.

### Diagnostic performance of the Panbio^™^ COVID-19 Ag rapid test in comparison with qRT-PCR

In this study, 31 (13.1%) of study participants tested positive for Panbio^™^ Ag-RDT and RT-PCR. A total of nine (22.5%) and three (1.5%) study participants were reported as false negative (Ag-RDT−/qRT-PCR+) and false positive (Ag-RDT+/qRT-PCR-) by Panbio^™^ Ag-RDT tests, respectively (Table 2).

**Table 2:** Comparison of performance of Panbio^™^ COVID-19 Ag Rapid Test with qRT-PCR test.

Panbio™ COVID-19 Ag Rapid Test Result	qRT-PCR test result		Total
	Negative (%)	Positive (%)	No. (%)
Negative	193 (98.5)	9 (22.5)	202 (85.6)
Positive	3 (1.5)	31 (77.5)	34 (14.4)
Total no. (%)	196 (83.0%)	40 (17.0)	236

Panbio^™^ COVID-19 Ag test had a sensitivity and specificity of 77.5 and 98.5%, respectively. The agreement between the two methods was 94.9% (kappa = 0.81, 95% CI: 0.7–0.9). The test had a PPV and NPV of 91.2 and 95.5%, respectively, considering the 17% prevalence of SARS-CoV-2 in the study area during the data collection period (Table 3).

**Table 3:** Estimation of performance of the Panbio™ COVID-19 Ag Rapid Test Device compared to qRT-PCR.

Statistics	Value (95% CI)
Sensitivity	77.5% (61.6–89.2%)
Specificity	98.5% (95.6–99.7%)
Positive predictive value	91.2% (76.9−97%)
Negative predictive value	95.5% (92.3–97.4%)

The sensitivity of Panbio™ COVID-19 Ag-RDT device was 94.4% (95% CI: 90.3–98.5%), 85.7% (95% CI: 80.3–91.1%), and 50% (95% CI: 34.3–65.7%) in a specimen collected from the patients with the duration of signs/symptoms 1–5 days, 1–7 days, and ≥ 7 days, respectively. The RDT had a sensitivity of 75% (95% CI: 57.80–87.9%) and a specificity of 98.9% (95% CI: 95.7–99.9%) in a specimen collected from adults (>18 years of age). However, the sensitivity of RDT increased to 100% (95% CI: 39.8–100%), but the specificity decreased to 96.8% (95% CI: 83.3–99.9%) in a specimen collected from study participants in the age group ≤18 years old. The kit had a sensitivity of 86.7% (95% CI: 59.5–98.3%) and a specificity of 100% (95% CI: 92.8–100.0%) when comparing study participants with a history of contact with confirmed SARS-CoV-2 patients or not. The sensitivity of the kit was higher among study participants who had contact with a household [90.0% (95% CI: 81.4–98.6%)] than those who did not (Table 4).

**Table 4:** Estimation of performance of the Panbio™ COVID-19 Ag Rapid Test Device compared to qRT-PCR by the duration of signs/symptoms and age groups of study participants.

Variable	Category	Panbio™ COVID-19 Ag Rapid Test Result	qRT-PCR test result	
			Neg no. (%)	Pos no. (%)
Duration of sign/symptoms (Days)	1–5 (*n* = 119)	Neg	99 (98.0)	1 (5.6)
		Pos	2 (2.0)	17 (94.4)
	1–7 (*n* = 160)	Neg	130 (98.5)	4 (14.3)
		Pos	2 (1.5)	24 (85.7)
	≥7 (*n* = 39)	Neg	30 (96.8)	4 (50.0)
		Pos	1 (3.2)	4 (50.0)
Age groups	≤18 (*n* = 35)	Neg	30 (96.8)	–
		Pos	1 (3.2)	4 (100%)
	>18 (*n* = 201)	Neg	163 (98.8)	9 (25.0)
		Pos	2 (1.2)	27 (75.0)
History of contact with confirmed SARS-COV-2 patients	Yes	Neg	49 (100)	2 (13.3)
		Pos	–	13 (86.7)
	No	Neg	144 (98)	7 (28)
		Pos	3 (2)	18 (72)
Source of contact	Household (*n* = 47)	Neg	37 (100)	1 (10.0)
		Pos	–	9 (90.0)
	Non-household (*n* = 17)	Neg	15 (100.)	1 (50.0)
		Pos	–	1 (50.0)

Pos: Positive; Neg: Negative.

The Ct values of NP samples with positive Panbio™ Ag-RDT ranged from 16.9 to 35.3 with a mean Ct value of 28.2 (±4.7), while the Ct values of samples reported as false-negative Panbio™ Ag-RDT ranged from 20.2 to 37 with the mean Ct value of 29.76(±5.7). The highest sensitivity of the Panbio™ COVID-19 Ag Rapid Test was obtained in those samples with low Ct-values of <20. However, the positivity of the Panbio™ COVID-19 Ag-RDT, when compared with 16 < Ct ≤25 (*n* = 11), 25 < Ct ≤30 (*n* = 12), 30 < Ct ≤37 (*n* = 17), had no significant Ct values (Pearson’s chi-square = 0.8 and *p* = 0.7) (Table 5).

**Table 5:** The positivity of Panbio™ Ag-RDT results according to the cycle threshold (Ct) value.

RT –PCR Ct value	Ag-RDT		
	Negative	Positive	% of positive
Ct 16–20 (*n* = 1)	0	1	100%
20 < Ct ≤25 (*n* = 10)	2	8	80%
25 < Ct ≤30 (*n* = 12)	2	10	83.3%
30 < Ct ≤37 (*n* = 17)	5	12	70.6%

RT-PCR: Real Time Reverse Transcription -Polymerase chain reaction; RDT: Rapid diagnostic tests RDT.

## Discussion

Adequate diagnostic testing for SARS-CoV-2 is a critical component of the overall prevention and control strategy for COVID-19. Rapid antigen diagnostic tests (RDT) can be an appropriate alternative to qRT-PCR for expanding testing capacity and reducing the burden on laboratories in many diagnostic settings. The ability to perform this test in patient care centers would simplify the process of testing, provide rapid results to the doctor, and also facilitate self-testing by individuals, thus improving the decision-making process and reducing pressure on healthcare providers (3, 4).

In this study, the Panbio™ tests had an overall sensitivity of 77.5% (61.6–89.2%). This was similar to the studies reported in Ethiopia (74.2%) (13), Valencia, Spain (79.6%) (14), Madrid, Spain (73.3%) (15), Switzerland (81%) (16), Brazil (84%) (17), Geneva University Hospital (85.5%) (18), Marseille, France (75.5%) (19), Sint Maarten, Dutch Caribbean (84%) (20), and Vienna (73.33%) (6). But it was lower than the sensitivity reports at Margalla Hospital, Taxila (94.3%), and Spain (90.5%), which might be due to all study participants having short-duration symptoms (21). However, the current finding is lower than the 91.4% sensitivity of the test reported by the manufacturers (10). The WHO guidelines require that SARS-CoV-2 RDTs demonstrate >80% sensitivity and ≥ 97% specificity compared with qRT-PCR. The Ag-RDTs should be prioritized for use in symptomatic individuals meeting the case definition for COVID-19 (4). In this study, the sensitivity of the Panbio™ test was high in samples collected from individuals with 1–5 days duration of COVID-19 symptoms (94.4%) (95% CI: 90.3–98.5%). This was similar to the 91.8 and 94.8% reports from Spain (22) and Geneva Hospital (18), respectively. However, the current study finding is higher than the two reports from Spain (80.4 and 85.3%) (14, 15).

In the current study, the sensitivity of Panbio decreased from 85.7% in the samples collected after 1–7 days of onset of signs and symptoms to 50% after more than 7 days. This was similar to 86.5 and 53.8% sensitivity reports on the samples collected from symptomatic patients with *<*7 days and ≥ 7 days, respectively, from Madrid, Spain (15). A similar decline in the sensitivity of the Panbio™ test with an increase in the number of days of signs and symptoms of COVID-19 was found in a study report from Geneva Hospital (18).

In this study, the sensitivity of the Panbio™ COVID-19 Ag Rapid Test was higher among study participants with the age <= 18 years (100%) than among > 18 years (75%). This is different from the study conducted in Spain, which found higher sensitivity among adults (82.6%) than among pediatrics (62.5%) (14). Another previous study found that the viral load in the respiratory tract was not significantly different by age (23). The difference might be due to the small number of pediatric participants in the study (14.8%), which might affect the proportion calculation.

The current test had higher sensitivity among contacts with households (90.0%) than non-household contact (50.0%). This finding was different from the report of 50.8 and 35.7% from Valencia, Spain, among asymptomatic households and non-households, respectively (24). Most of those contacts with signs and symptoms of COVID-19 in the current study were confirmed cases which can have serious impact on risk groups such as the elderly and those with chronic problems in their house. In general, it is very important to identify infected individuals who present with COVID-19 symptoms and individuals who have contact with infected individuals who might be responsible for the transmission of SARS-CoV-2 in the community.

In this study, the highest sensitivity (100%) of the Panbio™ COVID-19 Ag Rapid Test was obtained in those samples with Ct-values of <20. This was similar to the report from Geneva Hospital (98.4%) (18). However, the sensitivity decreased in those samples with threshold cycles (Ct) <25 (80%). This was different from the studies reported from Spain (99.5%) (22) and Geneva (95.5%) (18). The lowest sensitivity value in the current study was found for a specimen having 30 < Ct ≤37 (70.6%). This was higher than the 40.9% report from Geneva Hospital (18). This difference might be due to variations in the epidemiology of SARS-CoV-2 by geographical locality, sample size, viral copies of SARS-CoV-2, varying interpretation results based on the kit and others.

According to the World Health Organization (WHO) recommendations, Ag-RDTs perform best in individuals with high viral load, early in the course of infection, and in settings where SARS-CoV-2 prevalence is ≥5%. When there is no transmission or low transmission, the positive predictive value of Ag-RDTs will be low, and in such settings, qRT-PCR is preferable for first-line testing or confirmation of Ag-RDT positive results (4). During September, the prevalence of SARS-CoV-2 in Ethiopia was 7.9% (2) and 15.5% in the Harari Regional State (9).

The Panbio test’s current study had an overall specificity of 98.5% (95.6–99.7%). This is similar to the report studies conducted in Switzerland (99.1%) (16), Brazil (98%) (17), and Sint Maarten, Dutch Caribbean (99.9%) (20). This finding was slightly lower than the 100% report from Valencia, Spain (14), Ethiopia (13), and Geneva (18) and higher than a report from Marseille, France (94.9%) (19) and from Margalla Hospital, Taxila (37.9%) (21).

This study used the nasopharyngeal swab (NPS), which is considered the gold standard for the diagnosis of SARS-CoV-2 infection (25, 26). However, other samples like the nasal swab (NS) and saliva were often used due to their less invasive sampling method, higher tolerance, and more comfortable for individuals, offering new opportunities for SARS-CoV-2 testing strategies (27). The sensitivity of nasopharyngeal (NP) swabs in the current study (77.5%) was slightly lower than a study conducted on a nasal swab in Argentina (81.6%) (27) but higher than reports from Korea (64%) and Vienna (63.04%) (6). The specificity of Panbio™ tests from NP in the current study (98.5%) was slightly lower than a study conducted in Argentina and Korea (100.0%) (28, 29). Rapid Ag tests were reported to have stable sensitivity and specificity from nasal swab samples (6).

In this study, the Panbio™ rapid tests had a positive predictive value of 91.2% (95% CI: 76.9–97%) and a negative predictive value of 95.5% (95% CI: 92.3–97.4%). This is in agreement with studies conducted in Marseille, France and Sint Maarten, Dutch Caribbean and found positive predictive values of 95.6 and 91.7%, respectively. However, it is higher than the NPV report of 72.2% in Marseille, France and slightly lower than the NPV report found in Sint Maarten of the Dutch Caribbean (98%) (19, 20).

The above difference in Panbio performance might be due to the differences in viral load, prevalence or incidence of SARS-CoV-2, patient clinical characteristics and age, type of specimen processed, and duration of symptoms (7).

In the current study, the agreement between the two methods was 94.5% (kappa = 0.8, 95% CI: 0.7–0.9). This was similar to two studies reported from Spain (*k* = 0.87 and 0.90) (14, 22).

The study used a reasonable sample size and followed the strict SOPs for sample collection and examination with Panbio and RT-PCR by including both symptomatic and asymptomatic individuals. However, the study used self reported symptoms, and duration by patients which might have the evaluation RDT test. Viral culture had not been performed to assess the viability and infectivity of SARS-CoV-2 among those with SARS-CoV-2-positive individuals.

## Conclusion

In this study, the Panbio™ COVID-19 Ag Rapid Test Device had a sensitivity of 77.5%. The sensitivity of the test increased to ≥90% in the samples collected from patients with the sign and symptom duration of 1–5 days, age group of ≤18 years old, Ct-values of <20, and household source of contact. As a result, the Panbio™ COVID-19 Ag Rapid Test Device can be used in Ethiopian healthcare settings to diagnose symptomatic COVID-19 patients with a short clinical course and individual contact with infected individuals in the households. This study also recommends using qRT-PCR, especially for those with symptoms for more than 1 week or currently without symptoms.

### Limitations of the study

The small number of positive SARS-CoV-2 cases might not reflect the actual performance of the RDT, so it was better to evaluate the RDT in light of the result of qRT-PCR.

## Data availability statement

The original contributions presented in the study are included in the article/supplementary material, further inquiries can be directed to the corresponding author.

## Ethics statement

This study protocol was reviewed and approved by the Harari Heath Bureau’s Ethical and Research Review Board. Each study participant was given detailed information about the study, and written and signed consent was obtained from each study participant, as well as from their parents for children to participate in this study.

## Author contributions

DF, ZT, and MB conceptualized the study. AsA, AD, NS, KB, and AM supported testing at the health facility and supervised data collection. DF, MB, AbA, and AT conducted the qRT-PCR tests. DF, ZT, MB, and AAb conducted data validation, cleaning, and data analysis. ZT wrote the first manuscript. All authors participated in the development of the proposal for the study, read, reviewed, and edited the final manuscript.

## Conflict of interest

The authors declare that the research was conducted in the absence of any commercial or financial relationships that could be construed as a potential conflict of interest.

## Publisher’s note

All claims expressed in this article are solely those of the authors and do not necessarily represent those of their affiliated organizations, or those of the publisher, the editors and the reviewers. Any product that may be evaluated in this article, or claim that may be made by its manufacturer, is not guaranteed or endorsed by the publisher.
